# Weaver's historic accessible collection of synthetic dyes: a cheminformatics analysis†
†The views expressed in this paper are those of the authors and do not necessarily reflect the views or policies of the US Environmental Protection Agency. Mention of trade names or commercial products does not constitute endorsement or recommendation for use.
‡
‡Electronic supplementary information (ESI) available. See DOI: 10.1039/c7sc00567a



**DOI:** 10.1039/c7sc00567a

**Published:** 2017-04-07

**Authors:** Melaine A. Kuenemann, Malgorzata Szymczyk, Yufei Chen, Nadia Sultana, David Hinks, Harold S. Freeman, Antony J. Williams, Denis Fourches, Nelson R. Vinueza

**Affiliations:** a Department of Textile Engineering , Chemistry and Science , College of Textiles , North Carolina State University , Raleigh , NC 27695 , USA . Email: nrvinuez@ncsu.edu; b Department of Chemistry , Bioinformatics Research Center , College of Sciences , North Carolina State University , Raleigh , NC 27695 , USA . Email: dfourch@ncsu.edu; c National Center for Computational Toxicology , US EPA , Research Triangle Park , Durham , NC 27711 , USA . Email: williams.antony@epa.gov

## Abstract

We present the Max Weaver Dye Library, a collection of ∼98 000 vials of custom-made and largely sparingly water-soluble dyes. Two years ago, the Eastman Chemical Company donated the library to North Carolina State University. This unique collection of chemicals, housed in the College of Textiles, also includes tens of thousands of fabric samples dyed using some of the library's compounds. Although the collection lies at the core of hundreds of patented inventions, the overwhelming majority of this chemical treasure trove has never been published or shared outside of a small group of scientists. Thus, the goal of this donation was to make this chemical collection, and associated data, available to interested parties in the research community. To date, we have digitized a subset of 2700 dyes which allowed us to start the constitutional and structural analysis of the collection using cheminformatics approaches. Herein, we open the discussion regarding the research opportunities offered by this unique library.

## Abstract 2

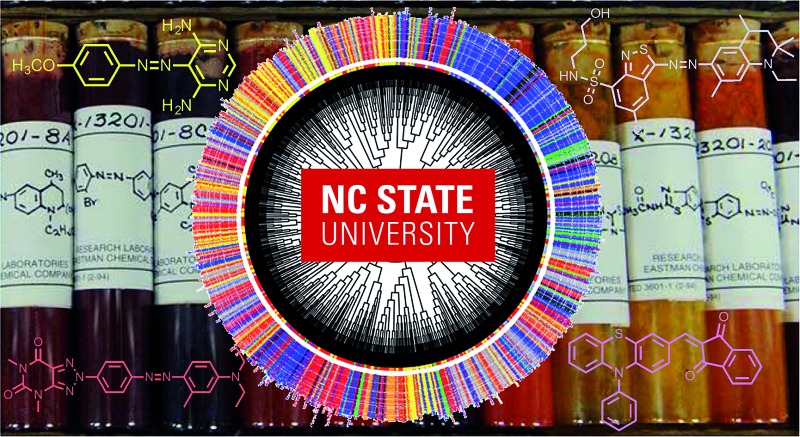
The Max Weaver Dye Library is presented to the scientific community with a cheminformatics approach to enhance research opportunities with this unique collection of ∼98 000 vials of custom-made dyes.

## 

Synthetic dyes are organic compounds possessing three essential characteristics: (1) one or more color bearing groups (*e.g.*, azo, anthraquinone, phthalocyanine, methine, nitro, triarylmethane); (2) ability to absorb light in the visible region (400–700 nm) of the electromagnetic spectrum; and (3) extended conjugation.^1^ Dyes are often classified based on their actual field of application (*e.g.*, food, drugs, cosmetics, textiles, photography, hair, lasers) and their molecular structure. Dyes are indispensable color additives in all of the aforementioned areas, especially textiles.

With colorants for cellulose acetate and poly(ethylene terephthalate) fibers in mind, Max Weaver and a small team of largely synthetic organic chemists created a remarkable Dye Library at Eastman Kodak Company over a 30 year period that began in the mid-1960s. When he simultaneously received the prestigious Olney Medal, given for outstanding achievement in the field of textile chemistry and the Henry E. Millson Award for invention, he stated that “new ideas were the life's blood for the researcher and that new ideas, and hence inventions, cannot be planned, scheduled, or directed. However, they can be encouraged and facilitated by an environment of free thought, hard work, and attention focused on the problems that need to be solved. Communication and discussion of ideas with fellow workers stimulate additional ideas. If not put to practice, even the best ideas are useless.” He also believed that the information needed by the textile dye chemist to exploit new ideas must be generated empirically, not theoretically, and that the dye chemist could, and seemed obligated to, prepare what seems to be an almost unlimited number of dyes for evaluation on various textiles. Thus, research on dyes for polyesters started in 1934 at Eastman and continued until the dyes business was sold to Ciba-Geigy in 1986. Table 1 summarizes the collection of dyes prepared and evaluated, to provide coverage of the entire visible spectrum on textiles, encompassing azo, anthraquinone, methine, *ortho*-nitrodiarylamine, and other substructures. Since these dyes were developed with the coloration of textile fibers in mind, their color rather than absorption spectra were reported. Thus, the library includes physical samples of textiles dyed with most of the colorants. A summary of the correlation of color and absorption spectra is provided in Table S1.†

**Table 1:** Number of textile dyes produced at Eastman through 1986

Color	Number of dyes
Yellow	14 979
Orange	11 076
Red	27 112
Blue	30 009
Brown	10 048
Black	921

In October 2000, Eastman initiated a search for a new home for the Dye Library, as its commercial interest in synthetic colorants was coming to an end. In response to a proposal from the College of Textiles at North Carolina State University, Eastman agreed to donate this treasure trove^2^ to an academic institution. The donation came with the challenge of endeavoring new uses for the group of chromogens arising from Max's initial vision and to find modern ways to share this information outside NC State.

As the initial step in sharing the library with chemists worldwide, we started building an electronic database containing the chemical structures of the dyes and using this as the foundation on which to add other data including chemical properties and analytical spectra. The digital database of dyes complements NC State's development of a dyed fiber database of automotive and other fabrics for use in criminal investigations^3,4^ bringing new statistical evidence to forensic fiber analysis and examination. This is significant because no comprehensive forensic dye database currently exists. Thus, the key goal of this paper is to present our current efforts regarding the development of this database and the numerous ways the scientific community could benefit from this Eastman's gift of Max's remarkable gift.

The development and analysis of a large database containing dye chemical structures and related information requires the use of appropriate cheminformatics tools. To date, out of 98 000 vials, approximately 2700 associated dye structures have been digitized into electronic format using a chemical structure drawing package. Prior to the cheminformatics analysis, we applied a chemical curation and standardization protocol.^5^ Overall, this resulted in a set of 2196 dyes encompassing nine different color families. Five color families (blue, red, yellow, orange, and purple) contained between 223 and 695 compounds, whereas four families (green, brown, white and black) had less than 100 compounds (Fig. 1). As expected (Table 1), we found blue to be the most common color with 695 unique dyes.

**Fig. 1:**
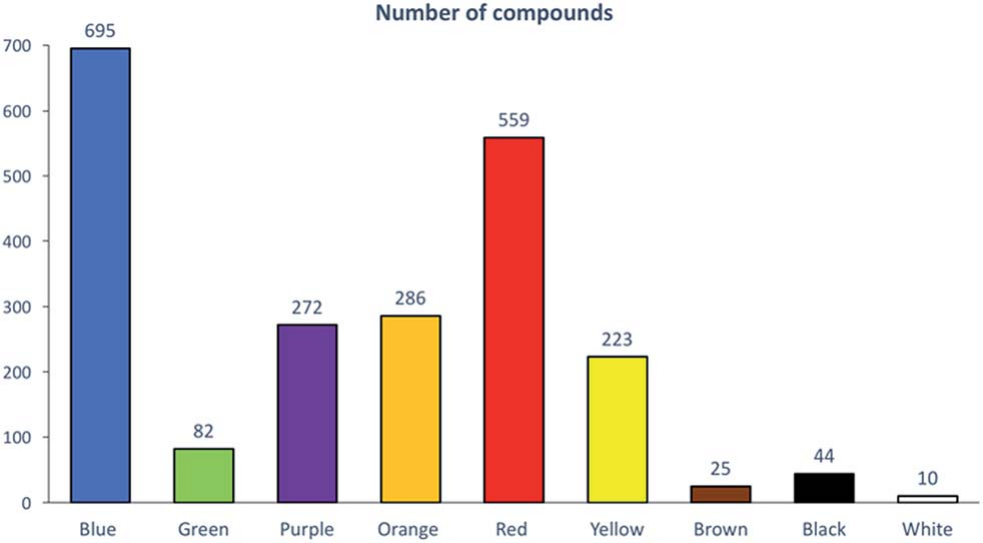
Distribution of the set of 2196 dyes according to their color as dyes for textile fibers.

Next, we evaluated how many dyes contain anthraquinone, azo, methine, nitro, triarylmethane and stilbene substructures, these substructures being well-known chromophores. As illustrated in Fig. 2, the azo substructure is the largest one present in our dataset with, 1637 compounds. Interestingly, we only found 2 triarylmethane and 25 stilbene containing compounds.

**Fig. 2:**
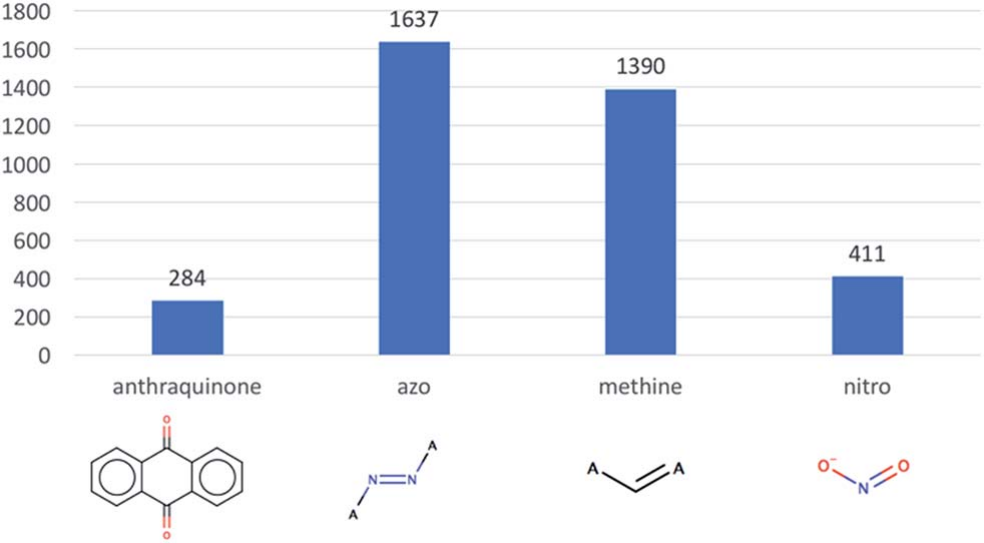
Distribution of different substructures present in the 2196 digitalized dyes.

We used KNIME^6^ to calculate 117 RDKIT descriptors to characterize key dyes' constitutional and structural properties for the dyes. We found that the dyes' average molecular weight (mean_MW_) was 439.7 g mol^–1^ ± 117.7, whereas their average hydrophobicity (mean_*S* log *P*_) was 5.03 ± 1.95 (Fig. 4A–C). Interestingly, a large portion of these dyes seemed to have properties potentially making them orally bioavailable: 38% of the collection passed Lipinski's rule of five^7^ with zero violations, whereas 71% and 91% of the remaining subset passed the rules with only one and two violations, respectively.

**Fig. 3:**
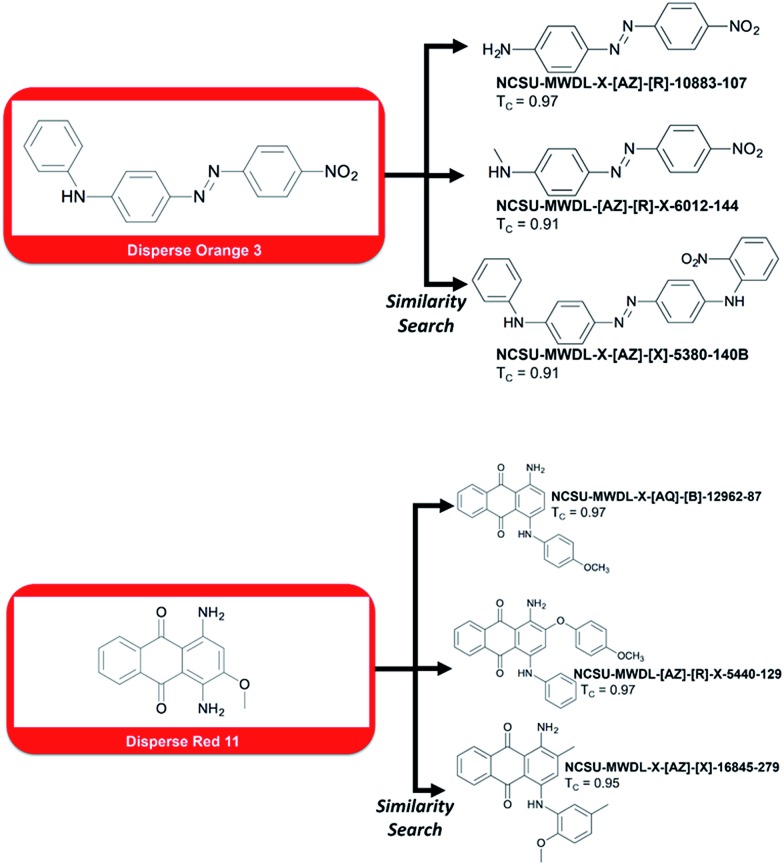
Top-3 dyes retrieved by similarity search using two probes, Disperse Orange 3 and Disperse Red 11, known to cause contact dermatitis.

**Fig. 4:**
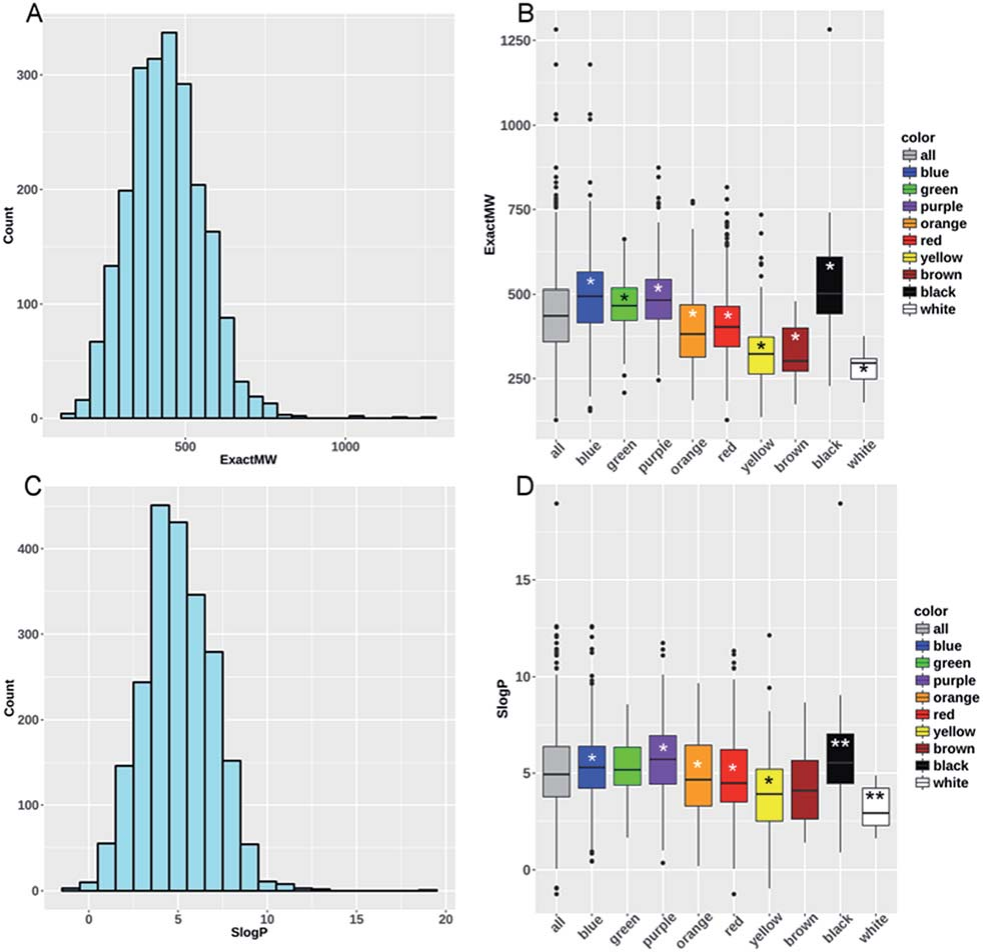
Distribution of dyes according to their molecular weight (A) and *S* log *P* (C). Boxplot of the MW (B) and *S* log *P* (D) are shown for all dyes as well as for each individual dye color. Stars on each boxplot represent the level of significance resulting from a pairwise comparison of the particular DYE color dataset *versus* all others colors from *moderately significant (0.01 < *P*-value < 0.05), **significant (0.001 < *P*-value < 0.01), to ***very significant (*P*-value < 0.001).

However, one could be interested in evaluating the detrimental biological effects caused by certain of these dyes. For instance, we searched the library for compounds that possess a similar structure to Disperse Orange 3 and Disperse Red 11, two compounds known to cause severe dermatitis.^8^

To do so, we ran a similarity search using MACCS fingerprints and the Tanimoto^9^ similarity coefficient (the closer to 1, the more structurally similar). Top-3 most similar dyes to Disperse Orange 3 and Disperse Red 11 are reported in Fig. 3. For example, compound NCSU-MWDL-X[AZ]-R-10883-107 from the Dye Library is also known as Armacel Orange GR, a compound recognized as a toxicant in 1979 by the US Environmental Protection Agency in the Toxic Substance Control Act.^10^ Thus, it could be interesting to screen the whole library of 98 000 chemical dyes against all the toxicants known to cause dermatitis or any other detrimental biological effects. Besides drug discovery, the Dye Library has an enormous potential for studies linked to chemical risk assessment, structure–toxicity relationships, and environmental impacts.

To help characterize the structural differences between the color families, we analyzed the distributions of the MW and *S* log *P* for each individual color subset (Fig. 4B and D). Yellow (mean_MW_ = 331.96 g mol^–1^ ± 90.4) and brown (mean_MW_ = 327.4 g mol^–1^ ± 84.5) compound subsets presented significantly lower mean values of MW and *S* log *P* compared to all of the other compounds (0.01 < *P*-value < 0.05, see Methods). On the contrary, purple and blue compound subsets presented significantly higher values for these two descriptors. The same analysis was conducted using the numbers of H-bond acceptors and donors, aromatic rings, the ratio of C sp^3^ carbon and the topological surface area (TPSA). Yellow compounds (mean_NumLipinskiHBA_ = 6.03 and mean_TPSA_ = 90.01 Å^2^) have significantly lower values for eight out of the nine descriptors (not true for the number of H-bond donors) compared to all of the other dyes contained within the dataset (see ESI Fig. S1–S5†). Conversely, blue compounds (mean_NumLipinskiHBA_ = 8.75 and mean_TPSA_ = 123.16 Å^2^) present significantly higher values for all nine descriptors except for the number of H-bond donors (ESI Table S2†). Obviously, these structural trends would need to be confirmed for the whole library of 98 000 dyes.

We then conducted an unsupervised hierarchical clustering based on the dyes' structural similarity. The resulting dendrograms using 66 uncorrelated RDKIT descriptors (Pearson's coefficient^11^ below 0.9), Euclidean distance, and a Ward linkage^12^ are given in Fig. 5.

**Fig. 5:**
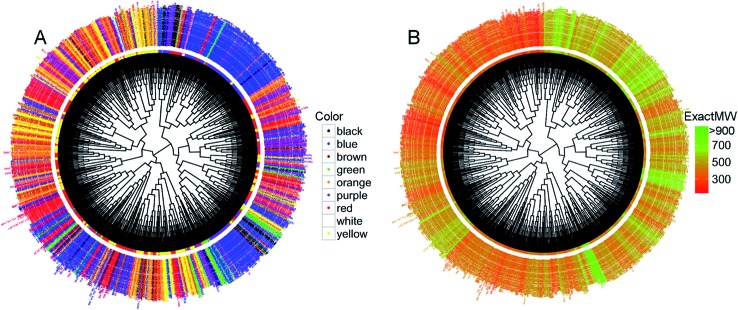
Circular dendrograms obtained from the hierarchical clustering of the set of 2196 dyes represented in RDKIT descriptor space. Compound nodes & names are colored according to their color (A) and their molecular weight (B).

With the dendrogram nodes colored according to the corresponding dye colors, it appears that the compounds not only form clusters with structurally similar molecules but also with the same color (Fig. 5A). For example, we observed a large cluster of 351 dyes containing 249 blue colored dyes (71%). There are other similar examples of smaller clusters with the vast majority of dyes belonging to the same color subset. When the dendrogram is colored according to the dyes' molecular weight (Fig. 5B), the large cluster of 249 blue dyes (on the upper right side of Fig. 5A) is also the same one containing dyes with high molecular weight (Fig. 5B). The same is true for the cluster on the upper left hand side that mainly contains yellow compounds, the latter presenting a very low molecular weight on the dendrogram of Fig. 5B. Overall, this clustering indicates that a large portion of dyes with high structural similarity share similar physicochemical properties and have the same or similar color too. Importantly, using different distance metrics and different linkages can change the results of the clustering. So, we also generated our hierarchical dendrogram changing the distance metrics and the linkage method (ESI Fig. S6–S9†). Despite these changes, we were able to retrieve similar or identical clusters: for example, the cluster containing a high number of blue dyes was retrieved whatever the parameters we used for the clustering.

However, we identified several pairs of dyes that have high structural similarity but different colors. Several examples of these “color cliffs” (in reference to “activity cliffs”^13,14^) are given in Fig. 6. In the first example (**6A**), the two azo compounds have the same molecular formula and the same absorption properties in solution (dimethylformamide), and are shown as rotamers in the dye collection. Interestingly, A1 is a yellow solid while A2 is orange, potentially due to differences in their crystal form. In the second example (**6B**), the two compounds possess the same molecular formula but red dye B1 has a chloro group in the *ortho*-position of the terminal phenylazo moiety, whereas B2 is reddish orange and has the chloro group in the *para* position. In the third example (**6C**), the two disubstituted anthraquinone compounds are constitutional isomers, having a pair of sulfanylbenzoic acid groups in the 1,4- *vs.* 1,8-positions leading to red and orange color dyes respectively.

**Fig. 6:**
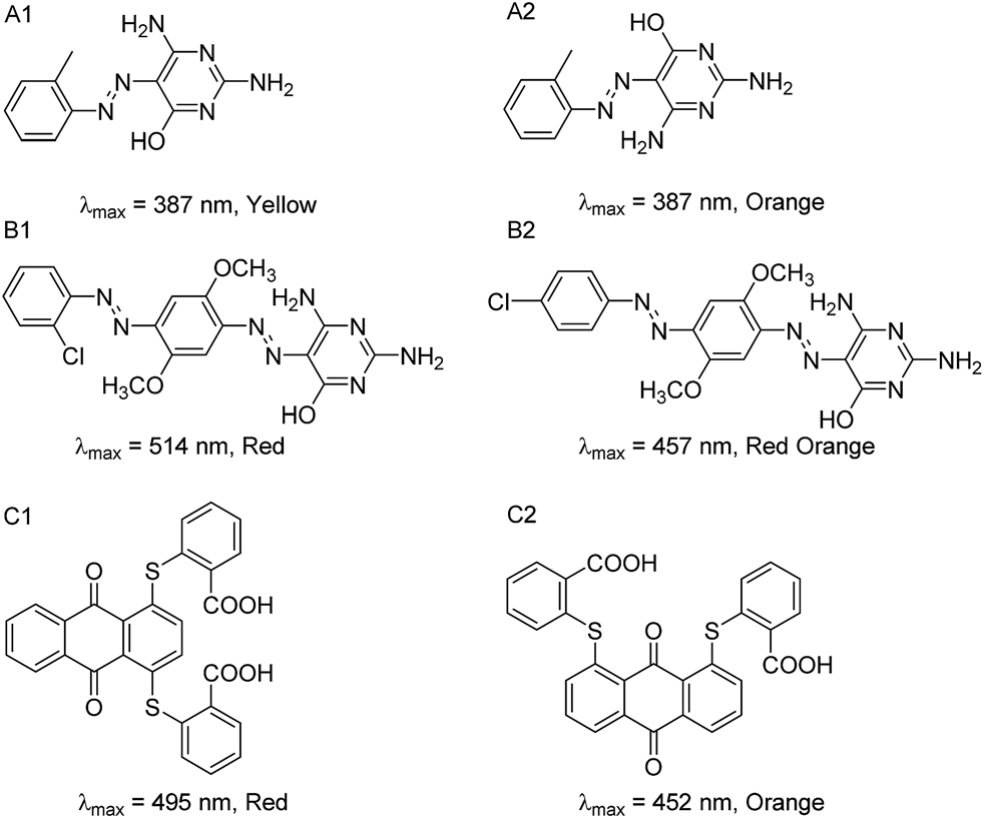
Three different pairs of dyes identified as potential “color cliffs”, *i.e.*, compounds with highly similar structures (Tanimoto coefficient ≥ 0.95 with MACCS fingerprints) but different colors.

Validation measurements to determine chemical formula accuracy were made using a randomly selected group of 74 dyes from the collection with the aid of high resolution mass spectrometry (MS). To illustrate the results, theoretical and experimental mass-to-charge (*m*/*z*) ratios are provided in Table 2 for dyes **A**, **B** and **C** (Fig. 6). The elemental composition for each dye is listed with the molecular structures (additional MS data are provided in ESI Table S3†)

**Table 2:** MS validation of dyes identified as “color cliffs”

Dye Library compound	Chemical formula	Theoretical *m*/*z* [M + H]^+^ ^*a*^	Experimental *m*/*z* [M + H]^+^ ^*a*^	Error (ppm)
**A1**	C_11_H_12_N_6_O	245.1145	245.1146	–0.41
**A2**	C_11_H_12_N_6_O	245.1145	245.1144	0.44
**B1**	C_18_H_17_N_8_O_3_Cl	429.1185	429.1188	–2.08
**B2**	C_18_H_17_N_8_O_3_Cl	429.1185	429.1189	–2.70
**C1**	C_28_H_16_S_2_O_6_	513.0461	513.0457	1.41
**C2**	C_28_H_16_S_2_O_6_	513.0461	513.0454	1.33

^*a*^Protonated molecules were selected for this analysis.

## Conclusions

The Dye Library offers untapped opportunities to (i) develop improved infrared absorption signatures for industrial use; (ii) create environmentally responsible dyes that can be applied to textiles, paper, packaging, cosmetics, hair coloring, and a host of other products; (iii) develop new dye sensitizers for solar cells; or (iv) take advantage of modern molecular modeling methods for prioritizing some dyes to be tested in anti-cancer and antibacterial assays. The latter interest was the genesis for the current paper. As the number of digitized dyes increases, further cheminformatics modeling will be possible with that larger dataset. As those results will be publicly shared in the form of peer-reviewed journal articles and conference papers, our ultimate goal is to digitize the entire Dye Library and add spectroscopic data. Various collaborations leading to the identification of dyes with potentially valuable chemical, physical, and biological properties are in progress and will be further expanded. In this regard, the late Max Weaver would be pleasantly surprised to learn that modern-day theoretical methods could hold the key to exploiting his ideas for new textile dyes for unforeseen applications in non-textile areas.

We wish to ensure that interested parties in the research community can obtain early access to the contents of the library. As a demonstration of feasibility, a subset of data associated with 150 representative dye structures has been made available online in two specific ways. The dye chemical structures have been deposited to the ChemSpider database under the data collection “NCSU Max Weaver Dye Library“ (www.chemspider.com/DatasourceDetails.aspx?id=900). An examination of the novelty of the dyes was performed by searching the first part of the InChI keys for the dyes across the ChemSpider database (which contains over 58 million unique chemicals as of March 2017). Nearly all (143) of the dyes were newly registered chemicals to the database while, 7 already had existing forms based on the InChI Key skeleton (ESI Fig. S10†). Interestingly, one particular dye had four related forms, based on tautomer and double bond types (E, Z and crossed-bond), already registered (; http://www.chemspider.com/Search.aspx?q=RWXSCGUFLZZHQD). When spectral data are available in the future, these will be associated as appropriate with each of the dye structures by depositing the spectra in JCAMP format (; www.jcamp-dx.org/protocols.html). The SDF file of dye chemical structures (including salt forms) has also been made available for download *via* the FigShare website (; https://figshare.com/articles/150_Analog_Max_Weaver_Dye_Library_Subset/4590250).

## Experimental

The elemental composition of the dyes was confirmed using an Agilent Technologies 6520 Accurate-Mass QTOF LC/MS (Agilent, Santa Barbara, CA) equipped with an ESI source, operated in negative or positive ion mode. Standard dye solutions were prepared by dissolving 1 mg of dye into 1 mL of methanol : water (60 : 40, v/v). The dye solutions were injected into the ESI source using a Harvard PhD 2000 Infusion syringe pump at a rate of 6 μL min^–1^. The operating conditions for ion formation consisted of nitrogen drying gas at a temperature of 350 °C and a rate of 10 L min^–1^, 35 psig nebulizer, 175 V fragmentor voltage, 65 V skimmer voltage, 750 V octopole voltage, 3500 V Vcap voltage, and 0.029 μA capillary current. The data acquisition and qualitative analysis were carried out by Agilent Mass Hunter Workstation Acquisition version B.05.00 and Qualitative Analysis Workstation Software version B.06.00. The UV-Visible spectra of dyes A–C were measured in dimethylformamide using an Agilent Technologies Cary 300 spectrometer.

### Computational details

All individual ChemDraw files were converted and concatenated into one file using MolConverter from the ChemAxon software suite (www.chemaxon.com). They were then standardized for the purpose of cheminformatics analysis: all salts and mixtures were flagged and removed solely for the purpose of the constitutional analysis conducted in this paper. Salts and mixtures are still included in the dye database. Structures were neutralized. Finally, all duplicates (352 compounds) were removed using the ISIDA-duplicates software.^15^ The evaluation of the number of anthraquinone, azo, methine, nitro, triarylmethane and stilbene substructures was performed using the KNIME node RDKIT Substructure Filter. Similarity to the compounds known to cause dermatitis (Disperse Orange 3 and Disperse Red 11) was conducted using MACCS fingerprints with KNIME and the Top-3 compounds based on Tanimoto similarity were kept. 117 2D chemical descriptors were calculated using the RDKIT descriptor^16^ calculation node in KNIME.^6^ Analysis of the mean values to distinguish the color group profiles for the seven selected descriptors (ExactMW, *S* log *P*, TPSA, NumLipinskiHBD, NumLipinskiHBA, FractionCsp3 and NumAromaticRings) were compared using an ANOVA analysis of variance. *Post hoc* comparisons were carried out using Tukey's test. In order to group similar compounds together, circular dendrograms were generated using the *ggtree* ^17^ R package (v3.2.4) with Euclidean distance and Ward linkage.^12^ Different linkage and distance metrics were tried to evaluate the stability of our clustering and are provided in ESI.† Only 66 uncorrelated RDKit descriptors (Pearson's coefficient below 0.9 (ref. 11)) were taken into account for clustering the dyes, not their actual color. Chemical similarity analysis to detect color cliffs was calculated with KNIME using MACCS fingerprints and compounds were considered as similar when their Tanimoto similarity^9^ was greater than 95%. In order to select the 150 representative dye structures, the Morgan and featMorgan fingerprints were calculated for each structure using KNIME. The representative molecules were then selected using the MaxMin algorithm with the RDKIT diversity picker node.
