# Supplementary material for: Weaver's historic accessible collection of synthetic dyes: a cheminformatics analysis
Source: Chem Sci. 2017 Apr 7;8(6):4334–9. doi: 10.1039/c7sc00567a (PMC5605791; doi:10.1039/c7sc00567a)
Supplement: Supplementary file 1 [file SC-008-C7SC00567A-s001.pdf]

Electronic Supplementary Material (ESI) for Chemical Science.
This journal is © The Royal Society of Chemistry 2017

Supporting Information

Weaver's Historic Accessible Collection of Synthetic Dyes:[1] A Cheminformatics Analysis

Melaine A. Kuenemann,[b] Malgorzata Szymczyk,[a] Yufei Chen,[a] Nadia Sultana,[a] David Hinks,[a] Harold S. Freeman,[a] Antony J. Williams,[c] Denis Fourches*[b] and Nelson R. Vinueza*[a]

[a]Department of Textile Engineering, Chemistry and Science, College of Textiles, North Carolina State, Raleigh, NC, 27695

[b]Department of Chemistry, Bioinformatics Research Center, College of Sciences, North Carolina State University, Raleigh, NC, 27695

[c] National Center for Computational Toxicology, US EPA, Research Triangle Park, Durham, NC, 27711

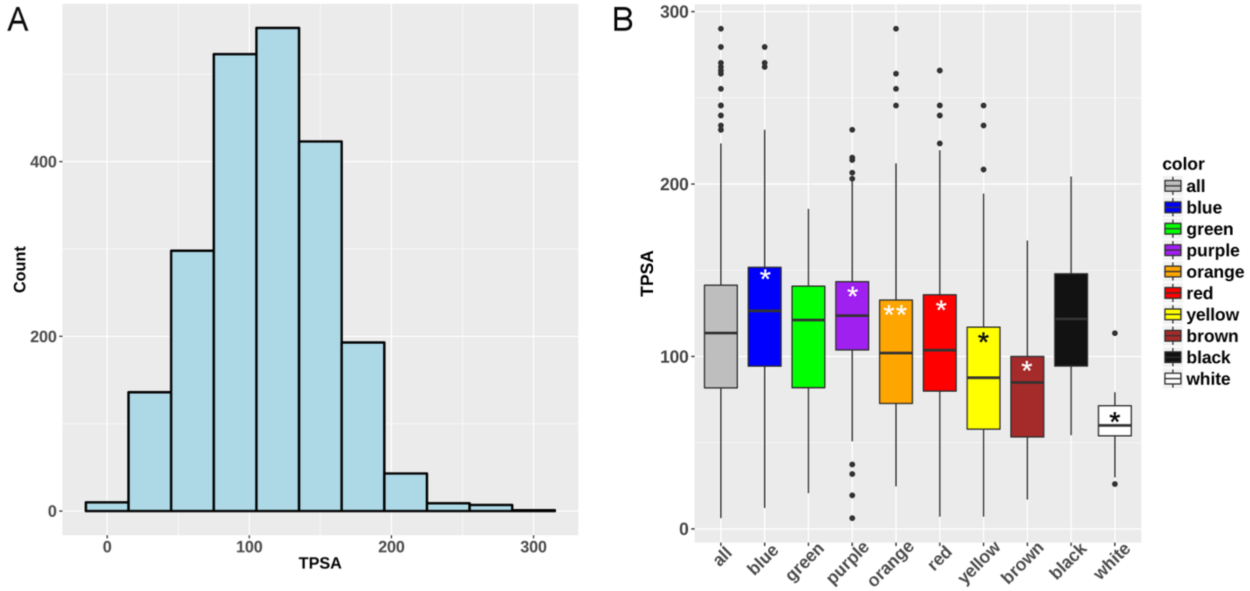

Figure S1. (A) Distribution and (B) boxplot of the total polar surface area (TPSA) for the set of 2,196 dyes. Stars on each boxplot represent the level of significance resulting from a pairwise comparison of a particular DYE color subset versus all other color subsets from *moderately significant (0.01 < P-value < 0.05), **significant (0.001 < P-value < 0.01), to ***very significant (P-value < 0.001).

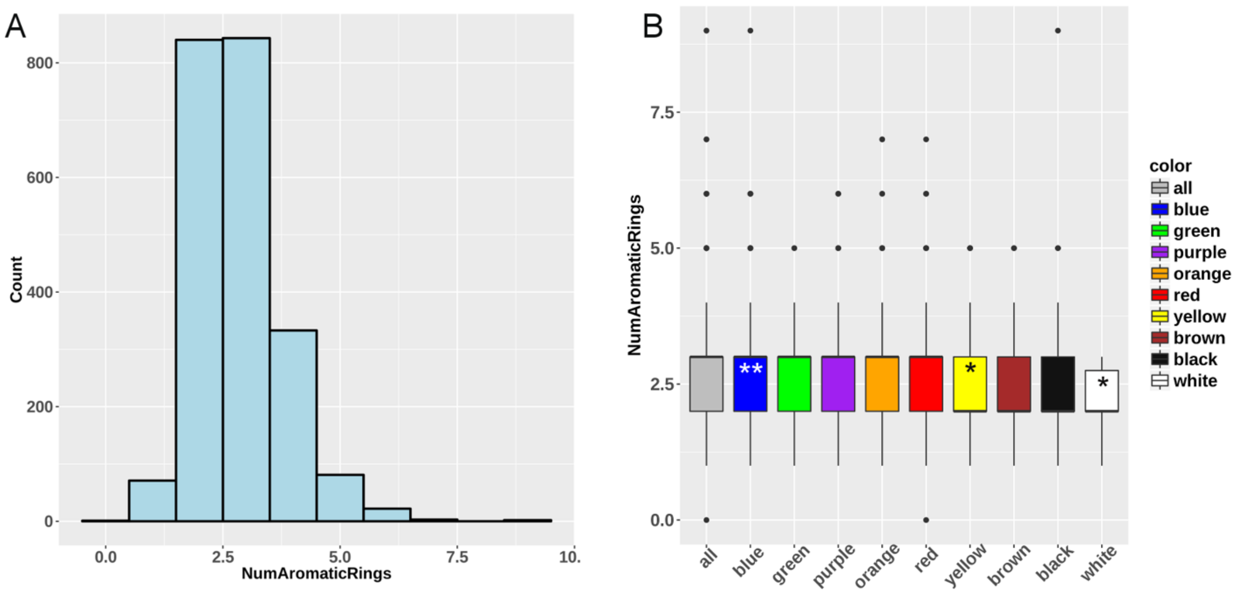

Figure S2. (A) Distribution and (B) boxplot of the intramolecular number of aromatic rings (NumAromaticRings) for the set of 2,196 dyes. Stars on each boxplot represent the level of significance resulting from a pairwise comparison of the DYE color dataset versus all others colors from *moderately significant (0.01 < P-value < 0.05), **significant (0.001 < P- value < 0.01), to ***very significant (P-value < 0.001).

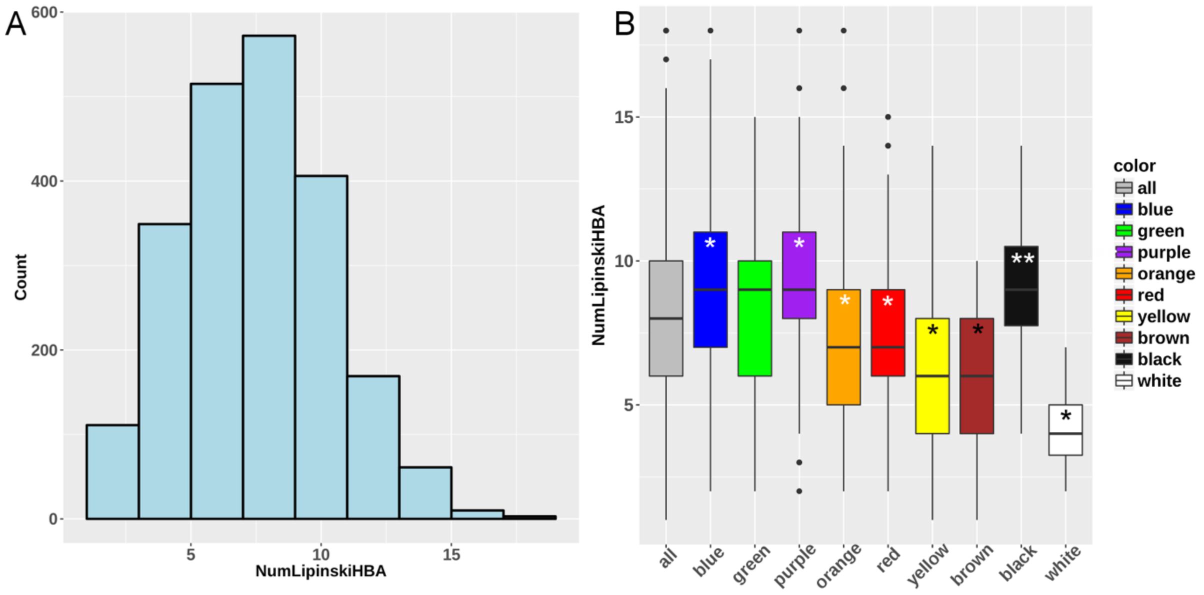

Figure S3. (A) Distribution and (B) boxplot of the number of H-bond acceptors (NumLipinskiHBA) for the set of 2,196 dyes. Stars on each boxplot represent the level of significance resulting from a pairwise comparison of the DYE color dataset versus all others colors from *moderately significant (0.01 < P-value < 0.05), **significant (0.001 < P- value < 0.01), to ***very significant (P-value < 0.001).

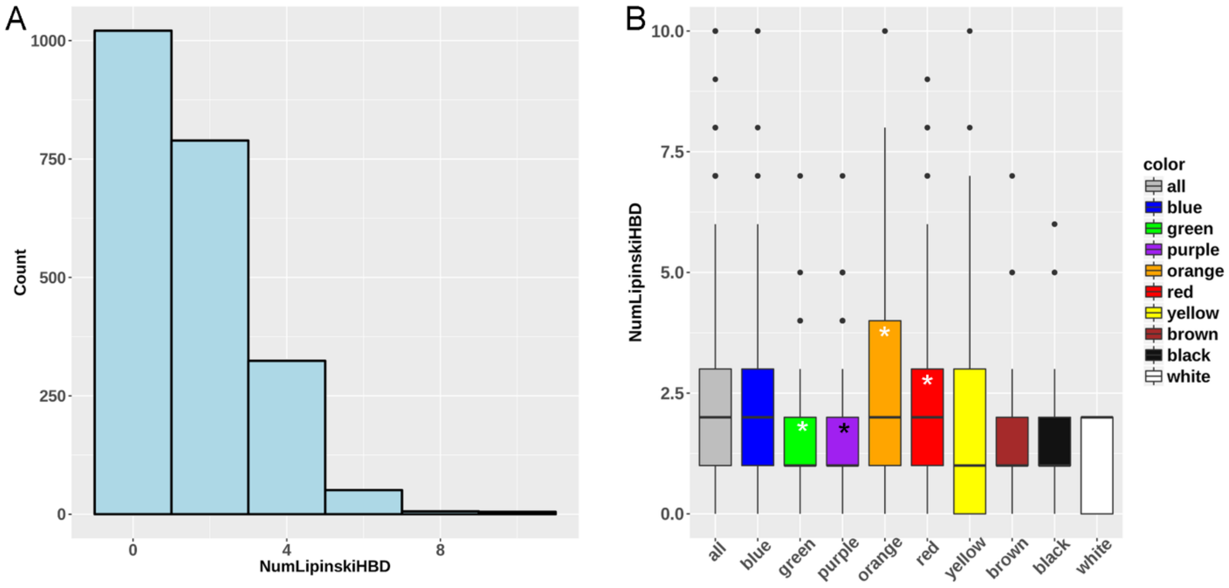

Figure S4. (A) Distribution and (B) boxplot of the number of H-bond donors (NumLipinskiHBD) for the set of 2,196 dyes. Stars on each boxplot represent the level of significance resulting from a pairwise comparison of the DYE color dataset versus all others colors from *moderately significant (0.01 < P-value < 0.05), **significant (0.001 < P- value < 0.01), to ***very significant (P-value < 0.001).

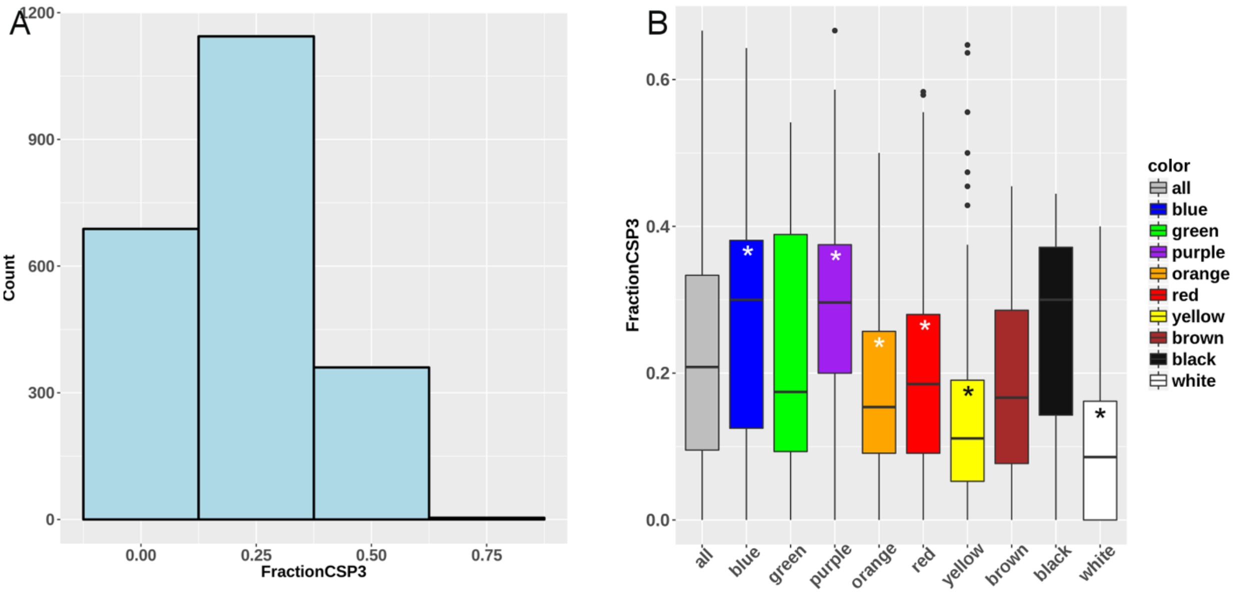

Figure S5. (A) Distribution and (B) boxplot of the ratio of Csp3 atom in the molecule for the set of 2,196 dyes. Stars on each boxplot represent the level of significance resulting from a pairwise comparison of the DYE color dataset versus all others colors from *moderately significant (0.01 < P-value < 0.05), **significant (0.001 < P- value < 0.01), to ***very significant (P-value < 0.001).

	total	*blue*	*green*	*purple*	*orange*	*red*	*yellow*	*brown*	*black*	*white*
**ExactMW**	439.71	493.89 (*)	467.61 (*)	488.37 (*)	393.87 (*)	413.26 (*)	331.96 (*)	327.43 (*)	525.53 (*)	287.25
**FractionCSP3**	0.22	0.27 (*)	0.23	0.29 (*)	0.18 (*)	0.19 (*)	0.13 (*)	0.18	0.26	0.1 (*)
**NumAromaticRings**	2.82	2.91 (**)	2.77	2.84	2.81	2.84	2.52 (*)	2.48	2.7	2.20 (*)
**NumLipinskiHBA**	7.98	8.75 (*)	8.34	9.12 (*)	7.37 (*)	7.58 (*)	6.03 (*)	5.68 (*)	9.11 (**)	4.10 (*)
**NumLipinskiHBD**	1.97	2.06	1.61 (*)	1.54 (*)	2.19 (*)	2.09 (*)	1.85	1.64	1.75	1.2
**TPSA**	112.84	123.16 (*)	113.7	123.36 (*)	105.91 (**)	108.89 (*)	90.01 (*)	81.35 (*)	123.36	61.81 (*)
**SlogP**	5.03	5.34 (*)	5.29	5.74 (*)	4.76 (*)	4.77 (*)	4.03 (*)	4.36	5.83 (**)	3.17 (**)

Table S1. Mean value for each descriptors, and each DYE color families. Stars on each row represent the level of significance resulting from a pairwise comparison of the DYE color dataset versus all others colors from *moderately significant (0.01 < P- value < 0.05), **significant (0.001 < P-value < 0.01), to ***very significant (P-value < 0.001).

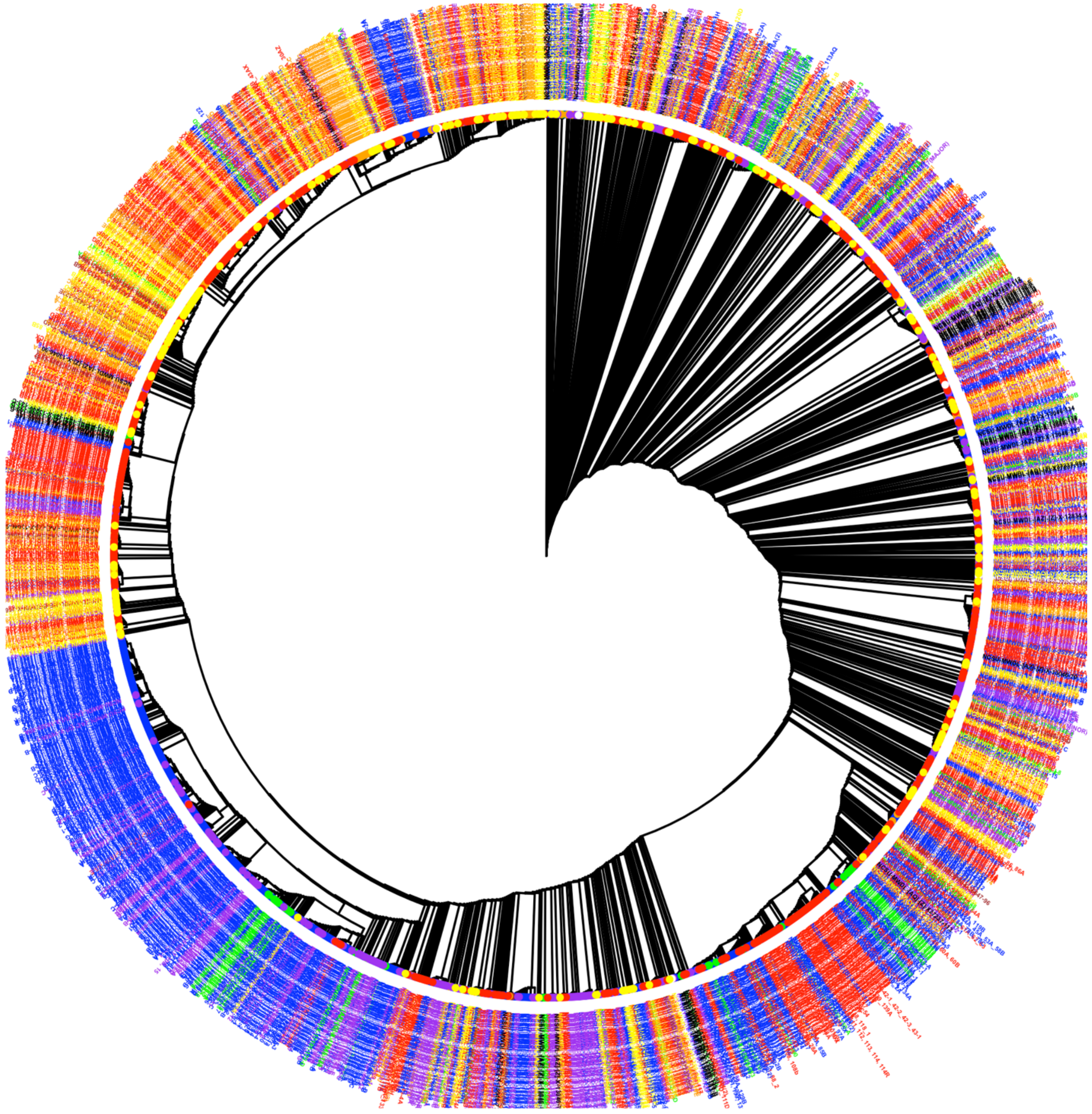

Figure S6. Circular dendrograms obtained from the hierarchical clustering of the set of 2,196 dyes represented in RDKIT descriptor space using Euclidian distance and single linkage. Compound nodes & names are colored according to their color

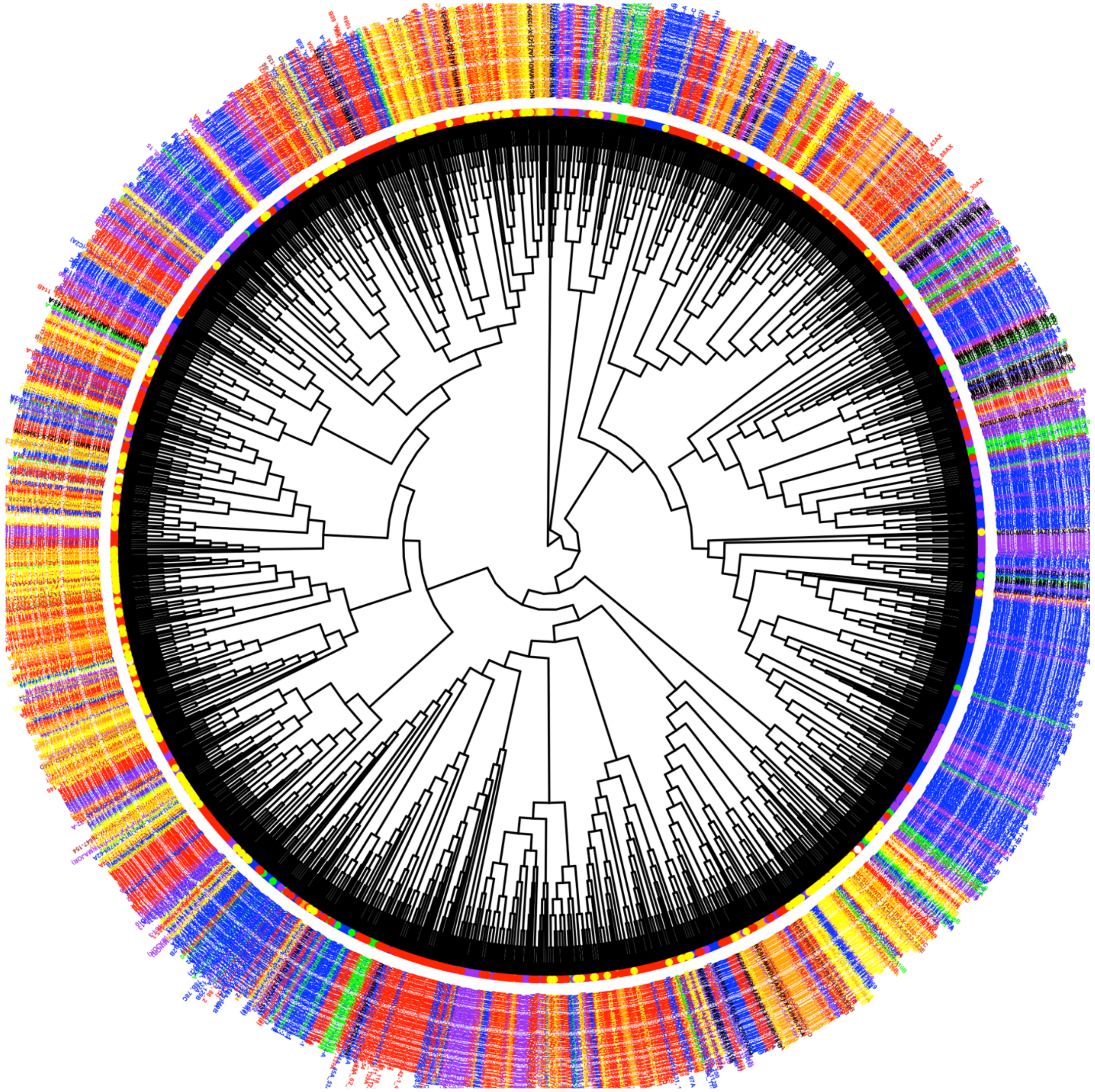

Figure S7. Circular dendrograms obtained from the hierarchical clustering of the set of 2,196 dyes represented in RDKIT descriptor space using Euclidian distance and complete linkage. Compound nodes & names are colored according to their color

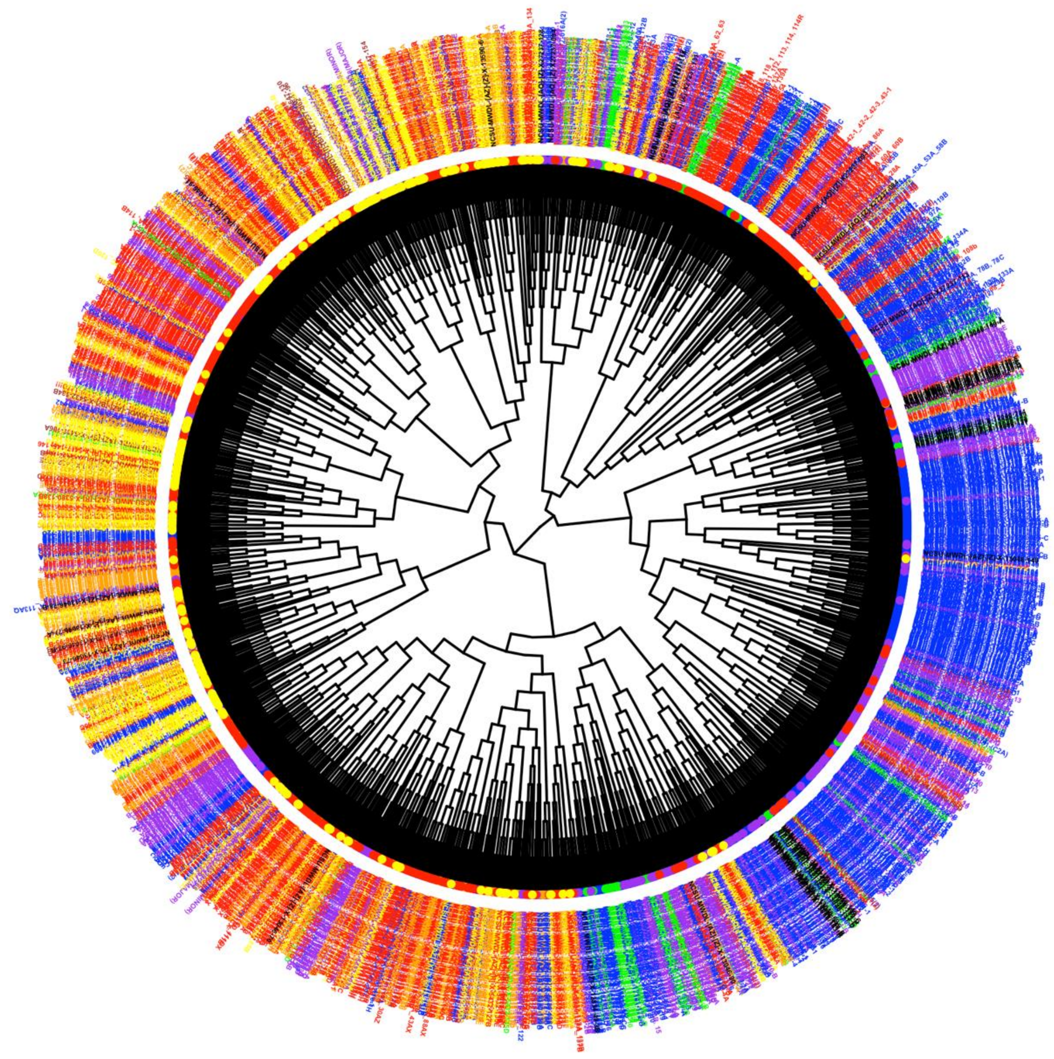

Figure S8. Circular dendrograms obtained from the hierarchical clustering of the set of 2,196 dyes represented in RDKIT descriptor space using Manhattan distance and Ward linkage. Compound nodes & names are colored according to their color

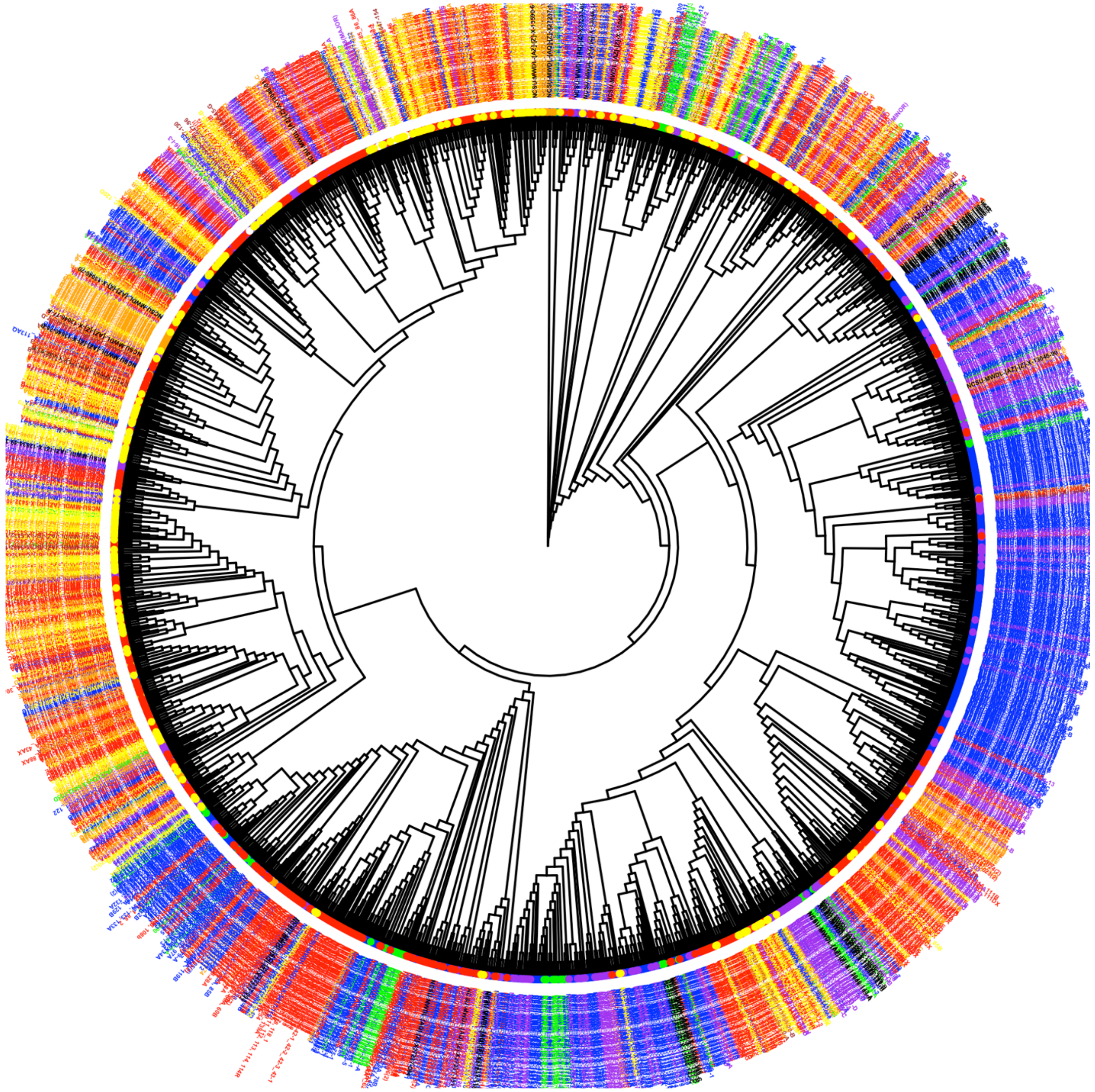

Figure S9. Circular dendrograms obtained from the hierarchical clustering of the set of 2,196 dyes represented in RDKIT descriptor space using Euclidian distance and average linkage. Compound nodes & names are colored according to their color

Table S2. NCSU Max Weaver Dye Library dyes analyzed by ESI-QTOF MS. All m/z experimental and theoretical values are from protonated molecules ($[M+H]^+$), except when indicated.

Dye Library I.D.	Formula	Theoretical m/z of $[M+H]^+$	Experimental m/z of $[M+H]^+$	Mass Error (ppm)
[AQ]-[B]-X-10469-71	$C_{26}H_{24}N_2O_4S$	461.1530	461.1516	-3.04
[AQ]-[B]-X-9525-9	$C_{26}H_{25}N_3O_5S$	492.1588	492.1573	-3.05
[AZ]-[R]-X-5380-143F	$C_{12}H_9N_5OS$	272.0601	272.0603	-0.62
[AQ]-[R]-X-23843-125	$C_{30}H_{36}Br_2N_2O_2$	615.1216	615.1229	-0.71
[AZ]-[O]-X-5432-55G	$C_{12}H_9N_7$	252.0992	252.1006	-2.76
[AQ]-[B]-X-23843-160	$C_{36}H_{34}N_2O_6S_2$	655.1931	655.1930	1.52
[AQ]-[B]-X-16145-251	$C_{21}H_{14}BrN_2O_5SNa$	$[M+Na]^+$ 530.9597	$[M+Na]^+$ 530.9609	-2.12
[AZ]-[B]-X-16926-56-A	$C_{24}H_{24}N_3O_6S_3K$	$[M+K]^+$ 624.0096	$[M+K]^+$ 624.0121	-4.11
[AZ]-[B]-X-16543-107-12	$C_{20}H_{24}N_4O_4S$	$[M+Na]^+$ 439.1410	$[M+Na]^+$ 439.1416	-1.84
[AZ]-[B]-X-16768-22-A	$C_{26}H_{24}N_8O_4$	513.1993	513.2008	-4.38
[AZ]-[B]-X-17945-99	$C_{27}H_{36}N_4O_3S$	497.2581	497.2589	-2.29
[AZ]-[B]-X-18213-25	$C_{27}H_{34}N_4O_6S_2Na$	598.1890	598.1900	-1.68
[AZ]-[B]-X-18329-116	$C_{19}H_{21}N_5OS$	368.1540	368.1548	-2.73
[ME]-[B]-X-21537-050	$C_{32}H_{28}N_2O_7$	$[M+Na]^+$ 575.1789	$[M+Na]^+$ 575.1822	-1.69
[AQ]-[B]-X-5496-28	$C_{25}H_{23}N_3O_3$	$[M+Na]^+$ 436.1632	$[M+Na]^+$ 436.1637	-2.06
[AZ]-[X]-X-5432-63-K	$C_{23}H_{20}N_8O$	425.1833	425.1829	0.81
[AQ]-[R]-X-27237-078	$C_{18}H_{12}N_4O_4S$	381.0652	381.0469	1.34
[AQ]-[B]-X-9525-15	$C_{28}H_{22}ClN_3O_4S$	532.1092	532.1078	-2.63
[AQ]-[B]-X-10469-110	$C_{20}H_{21}N_3O_6S$	432.1224	432.1209	-3.47

**[AZ]-[X]-X-6012-147D**	**$C_{25}H_{27}Cl_2N_5O_4$**	**532.1513**	**532.1511**	**0.75**
**[AQ]-[B]-X-9525-13**	**$C_{28}H_{23}N_3O_4S$**	**498.1482**	**498.1464**	**-3.61**
**[AQ]-[B]-X-9525-60**	**$C_{32}H_{33}N_3O_8S$**	**620.2061**	**620.2071**	**1.61**
**[AQ]-[B]-X-9525-102**	**$C_{22}H_{17}BrN_2O_3$**	**437.0495**	**437.0478**	**-3.89**
**[AQ]-[B]-X-10161-21**	**$C_{26}H_{18}N_2O_2S$**	**423.1162**	**423.1173**	**2.60**
**[AQ]-[B]-X-10161-70**	**$C_{26}H_{17}N_3O_4S$**	**468.1013**	**468.0997**	**-3.42**
**[AQ]-[B]-X-10161-91**	**$C_{26}H_{24}N_2O_4S_2$**	**493.1250**	**493.1237**	**-2.64**
**[AQ]-[B]-X-9525-10**	**$C_{27}H_{27}N_3O_5S$**	**506.1744**	**506.1734**	**-1.98**
**[AQ]-[B]-X-10469-103**	**$C_{22}H_{25}N_3O_5S$**	**444.1588**	**444.1572**	**-3.60**
**[AQ]-[B]-X-9525-51**	**$C_{31}H_{31}N_3O_9S$**	**622.1854**	**622.1823**	**-4.98**
**[ME]-[O]-X-26647-194**	**$C_{24}H_{22}Cl_2N_2O_6$**	**$[M+Na]^+$ 527.0747**	**$[M+Na]^+$ 527.0739**	**1.94**
**[ME]-[R]-X-21537-005**	**$C_{30}H_{28}SN_6O_6$**	**$[M+Na]^+$ 623.1683**	**$[M+Na]^+$ 623.1705**	**-4.15**
**[AZ]-[R]-X-13046-68**	**$C_{19}H_{21}N_5O_3S$**	**400.1438**	**400.1448**	**-2.14**
**[X]-[B]-X-5417-88**	**$C_{25}H_{21}ClN_2$**	**$[M-Cl]^+$ 349.1699**	**$[M-Cl]^+$ 349.1691**	**2.3**
**[AQ]-[B]-X-9525-71**	**$C_{28}H_{29}N_3O_5S$**	**520.1901**	**520.1885**	**-3.08**
**[AQ]-[B]-X-9525-14**	**$C_{28}H_{31}N_3O_5S$**	**534.2057**	**534.2038**	**-3.56**
**[AZ]-[B]-X-5380-100D**	**$C_{26}H_{27}N_4OSI$**	**$[M-I]^+$ 443.1900**	**$[M-I]^+$ 443.1918**	**-3.06**
**[AQ]-[B]-X-9525-11**	**$C_{27}H_{27}N_3O_5S$**	**506.1744**	**506.1725**	**-3.75**
**[X]-[Y]-X-732-85**	**$C_{29}H_{20}O$**	**385.1587**	**385.1580**	**2.18**
**[X]-[Y]-X-732-91C**	**$C_{21}H_{17}BrN_2S_2$**	**[M-Br]+ 361.0828**	**[M-Br]+ 361.0828**	**1.59**
**[AZ]-[B]-X-16181-205-7**	**$C_{20}H_{20}N_4O_4S_2$**	**445.0999**	**445.0984**	**3.33**
**[AQ]-[B]-X-9525-16**	**$C_{29}H_{25}N_3O_4S$**	**512.1639**	**512.1624**	**-2.93**
**[Ir]-[W]-X-25380-49**	**$C_{20}H_{14}N_2O_5$**	**363.0975**	**363.0974**	**0.52**

**[Ir]-[Y]-X-25380-174**	**$C_{16}H_9N_3S_2$**	**$[M+Na]^+$ 330.0130**	**$[M+Na]^+$ 330.0111**	**4.73**
**[ME]-[Y]-X-25380-113**	**$C_{22}H_{14}N_2OS_2$**	**387.0620**	**387.0618**	**0.51**
**[AQ]-[B]-X-9525-12**	**$C_{28}H_{29}N_3O_5S$**	**520.1901**	**520.1881**	**-3.84**
**[AZ]-[O]-X-13046-78-D**	**$C_{31}H_{28}N_6O$**	**501.2397**	**501.2409**	**-2.77**
**[AZ]-[Y]-X-13046-124-B**	**$C_{17}H_9Cl_3N_4S$**	**406.9686**	**406.9701**	**-3.35**
**[AZ]-[O]-13046-108-A**	**$C_{20}H_{12}Cl_3N_3$**	**400.0170**	**400.0180**	**-2.44**
**[AQ]-[B]-X-9525-103**	**$C_{23}H_{19}BrN_2O_4$**	**467.0601**	**467.0582**	**-4.01**
**[ME-[B]-X-5417-88**	**$C_{25}H_{21}N_2Cl$**	**$[M-Cl]^+$ 349.1699**	**$[M-Cl]^+$ 349.1698**	**0.59**
**[AZ]-[R]-X-6012-129**	**$C_{16}H_{18}N_4O$**	**283.1553**	**283.1552**	**0.67**
**[AZ]-[R]-X-5432-134-A**	**$C_{11}H_{11}N_5$**	**214.1087**	**214.1083**	**1.77**
**[AQ]-[B]-X-9525-56**	**$C_{27}H_{27}N_3O_7S$**	**538.1642**	**538.1653**	**2.04**
**[AZ]-[O]-X-5432-26-C**	**$C_{23}H_{19}N_3O_4$**	**402.1448**	**402.1441**	**1.36**
**[AZ]-[O]-X-5432-55-G**	**$C_{12}H_9N_7$**	**252.0992**	**252.0987**	**2.04**
**[AQ]-[B]-X-5380-134-B**	**$C_{28}H_{23}N_3O_{10}S$**	**$[M-CH_3OSO_4]^+$ 482.1347**	**$[M-CH_3SO_4]^+$ 482.1362**	**-4.01**
**[AZ]-[R]-X-5380-112-A**	**$C_{16}H_{14}N_5S_2I$**	**$[M-I]^+$ 340.0685**	**$[M-I]^+$ 340.0688**	**0.02**
**[AZ]-[Y]-X-13480-62-A**	**$C_{14}H_9ClN_4O_3$**	**317.0436**	**317.0429**	**1.38**
**[Ir]-[Y]-X-732-100-A**	**$C_{22}H_{15}N_3$**	**322.1339**	**322.1331**	**2.42**
**[Ir]-[Y]-X-732-100-E**	**$C_{22}H_{14}N_2O_2$**	**339.1128**	**339.1122**	**1.68**
**[Ir]-[Y]-X-732-100-F**	**$C_{22}H_{12}N_2S_2$**	**369.0515**	**369.0509**	**1.39**
**[Ir]-[Y]-X-732-100-G**	**$C_{20}H_{16}N_2O_2S$**	**349.1005**	**349.0995**	**3.55**
**[ME]-[Y]-X-732-85**	**$C_{29}H_{20}O$**	**385.1587**	**385.1576**	**2.91**
**[Ir]-[Y]-X-732-90-A**	**$C_{19}H_{16}N_2O_3$**	**321.1234**	**321.1231**	**1.00**

**[Ir]-[Y]-X-732-90-B**	**$C_5H_7N_5O$**	**154.0723**	**154.0719**	**2.9**
**[Ir]-[Y]-X-732-91-C**	**$C_{21}H_{17}N_2S_2Br$**	**$[M-Br]^+$ 361.0828**	**$[M-Br]^+$ 361.0843**	**-2.5**
**[AZ]-[Y]-X-732-92-A**	**$C_{17}H_{14}N_2O$**	**263.1179**	**263.1173**	**2.77**
**[ME]-[Y]-X-732-92-B**	**$C_{20}H_{21}N_3O$**	**320.1757**	**320.1752**	**2.18**
**[AZ]-[G]-X-15557-269**	**$C_{35}H_{39}N_7O_5$**	**638.3065**	**638.3069**	**0.96**

Dye purity

Individual stock solutions of 1000 µg/mL for the reference compounds were prepared in acetonitrile. Working solutions of 30 µg/mL were prepared by dilution with acetonitrile.

Liquid Chromatography was performed on a ZORBAX C_{18} column (150×2.1 mm), fitted with a guard column with identical packing material (4 × 2.0 mm) (Agilent). The column oven was maintained at 45 °C, and 2 µL of each sample was injected. Gradient elution with (A) 0.1% formic acid in water, and (B) acetonitrile, at a flow rate of 0.5 mL/min, was applied. The initial gradient conditions were 40% B, increasing to 45% B in 0.5 min, with a final composition of 90% B in 5 min. The column was flushed for 5 min at 90% B. Initial gradient conditions were reestablished in 5 min, and the column was equilibrated for an additional 2 min.

Table S3. HPLC analysis of purity of selected dyes

**Dye Name**	**Purity, %**
A1	96.32
A2	96.00
B1	91.98
B2	97.89
C1	100.00
C2	100.00
[AQ]-[B]-X-10161-21	96.74
[AQ]-[B]-X-10161-70	76.50
[AQ]-[B]-X-9525-14	63.48
[AQ]-[B]-X-9525-9	83.85

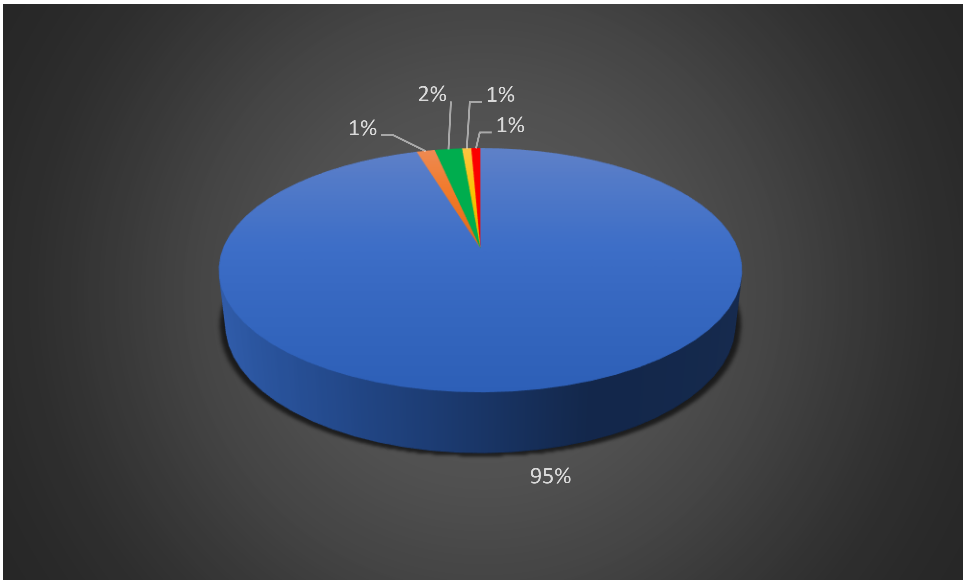

Figure S10. A pie chart showing the distribution of frequency of InChI skeleton distribution (e.g. 2% of the set has 3 equivalent molecular skeletons based on a search of the first part of the InChI Key search, thereby differing in either stereochemistry, charge, tautomer or isotope labeling)
